# "If it ain't broke, don't fix it": a commentary on the positive-negative results of the ACCORD Lipid study

**DOI:** 10.1186/1475-2840-9-24

**Published:** 2010-06-15

**Authors:** Alexander Tenenbaum, Enrique Z Fisman

**Affiliations:** 1 Cardiac Rehabilitation Institute, Sheba Medical Center, 52621 Tel-Hashomer, Israel; 2 Sackler Faculty of Medicine, Tel-Aviv University, 69978 Tel-Aviv, Israel; 3 Cardiovascular Diabetology Research Foundation, 58484 Holon, Israel

## Abstract

Even using intensive statin monotherapy, many patients fail to achieve all the desired lipid goals and remain at high residual risk of cardiovascular events. In view of the still unproven decisively intensive "statin as monotherapy" strategy and "residual risk" concept, it is logical to ask whether other strategies, particularly fibrate/statin combination therapy, could be more beneficial and safer. A clear benefit of fibrate monotherapy did emerge previously among patients with atherogenic dyslipidemia (particularly high triglycerides and low high density lipoprotein cholesterol [HDL-C]) typically present in the metabolic syndrome and type 2 diabetes. In contrast, in patients without atherogenic dyslipidemia this favorable effect was not demonstrated.

The Action to Control Cardiovascular Risk in Diabetes (ACCORD) study investigated whether combination therapy with a statin plus a fibrate, as compared with statin monotherapy, would reduce the risk of cardiovascular disease in patients with type 2 diabetes mellitus. However, relevant patients with atherogenic dyslipidemia represented less than 17 percent of the ACCORD Lipid population (941 out of 5518 patients). In this prespecified subgroup, the patients benefited from fenofibrate therapy in addition to simvastatin similar to the previous "fibrate's as monotherapy" trials: the primary outcome rate was 12.4% in the fenofibrate group, versus 17.3% in the placebo group (28% crude HR reduction, CI less than1, e.g. statistically significant findings). Among all other 4548 patients without atherogenic dyslipidemia such rates were 10.1% in both fenofibrate and placebo study groups. Authors concluded that in the overall cohort of patients the combination of fenofibrate and simvastatin did not reduce the rate of the cardiovascular events as compared with simvastatin alone. Thus, their results do not support the routine use of combination therapy with fenofibrate and simvastatin to reduce cardiovascular risk in the general patients with type 2 diabetes. A recent large meta-analysis regarding effects of fibrates on cardiovascular outcomes noted greater effect sizes in trials that recorded a higher mean baseline triglyceride concentration (p = 0.030). As expected, in a so called "general population", reflecting a blend of effects in patients with and without atherogenic dyslipidemia, a mean "diluted" effect of fibrate therapy was reduced, but still producing a significant 10% relative risk (RR) decrease in major cardiovascular events (p = 0.048) and a 13% RR reduction for coronary events (p < 0.0001).

It should be pinpointed that the epidemiological characteristics of the ACCORD Lipid study depart from those seen in real clinical practice: among people with type 2 diabetes, there is a high prevalence of atherogenic dyslipidemia and metabolic syndrome. For example, an analysis of NHANES III data in adults aged ≥50 years showed that approximately 86% of patients with type 2 diabetes also had the metabolic syndrome. Therefore, an importand finding of ACCORD Lipid study was the observation that fibrates may lead to cardiovascular risk reduction in patients with atherogenic dyslipidemia not only as monotherapy but in combination with statins as well.

In conclusion, in patients with atherogenic dyslipidemia (high triglycerides and low HDL-C, fibrates -- either as monotherapy or combined with statins - were associated with reduced risk of cardiovascular events. In patients without dyslipidemia this favorable effect - as expected - was absent.

## Introduction

### The ACCORD Lipid study: Brief formal overview

The Action to Control Cardiovascular Risk in Diabetes (ACCORD) study investigated whether combination therapy with a statin plus a fibrate, as compared with statin monotherapy, would reduce the risk of cardiovascular disease in patients with type 2 diabetes mellitus who were at high risk for cardiovascular disease [1]. In this trial 5518 patients with type 2 diabetes were randomly assigned to receive simvastatin plus fenofibrate or simvastatin alone (simvastatin plus placebo). The primary outcome was the first occurrence of nonfatal myocardial infarction, nonfatal stroke, or death from cardiovascular causes. The mean follow-up was 4.7 years. The annual rate of the primary outcome was 2.2% in the fenofibrate group and 2.4% in the placebo group (hazard ratio in the fenofibrate group, 0.92; 95% confidence interval [CI], 0.79 to 1.08; P = 0.32). There were also no significant differences between the two study groups with respect to any secondary outcome. Annual rates of death were 1.5% in the fenofibrate group and 1.6% in the placebo group (hazard ratio, 0.91; 95% CI, 0.75 to 1.10; P = 0.33). Prespecified subgroup analysis suggested a possible interaction according to lipid subgroup, with a possible benefit for patients with both a high baseline triglyceride level and a low baseline level of high-density lipoprotein cholesterol (P = 0.057 for interaction). Authors concluded that in the overall cohort of patients the combination of fenofibrate and simvastatin did not reduce the rate of the cardiovascular events as compared with simvastatin alone. Thus, these results do not support the routine use of combination therapy with fenofibrate and simvastatin to reduce cardiovascular risk in the the general patient with type 2 diabetes.

Despite the aforementioned conclusion, an in-depth examination of the ACCORD study itself and of several other mainstay trials concerning the pharmacological management of dyslipidemia, yields a completely different clinical picture. Namely, fibrates -- alone or combined with statins -- emerge as a proficient therapeutic tool leading to an improved cardiovascular outcome. This appraisal is depicted in the present commentary.

## Discussion

### Deadlock of the "statins as monotherapy" strategy

Even using intensive statin monotherapy, many patients with atherogenic dyslipidemia fail to achieve all the desired lipid goals and remain at high residual risk of cardiovascular events [2]. In addition, the prescription of statins in high doses may have important limitations in daily clinical practice: compared with low-dose therapy, intensive statin therapy has been associated with increased incidence of discontinuation, hepatotoxicity and myalgia [3]. Moreover, the incidence of side effects with intensive statin therapy in clinical practice might be higher than the figures reported in clinical trials as a result of the careful selection of patients (such as the exclusion of patients with known previous intolerance to statins). Observational studies suggest that muscle-related symptoms can be frequent in patients on statins; for example, they have been registered in 18.2% of patients receiving simvastatin [4].

Direct testing of varying degrees of low density lipoprotein cholesterol (LDL-C) lowering by using of active comparators (statin vs. statin) has been tested in 4 large outcomes trials[5-8]: PROVE IT--TIMI 22, A to Z, TNT and IDEAL. The fifth and largest of the trials [9] comparing intensive vs. standard-dose statins therapy, the Study of the Effectiveness of Additional Reductions in Cholesterol and Homocysteine (SEARCH), was expected to report its final conclusions in 2008 [9], but mysteriously disappeared from the scientific horizons (suggestively due to negative results).

The 'scores' of these trials where 'positive' means 'in favor of intensive LDL-C-lowering strategy using statins as monotherapy' are as follows:

1. PROVE IT--TIMI 22: high-dose strong atorvastatin (80 mg/day) demonstrated a modest 16% relative reduction in the risk of death and major cardiovascular events vs. medium-dose gentle pravavastatin (80 mg/day), which was observed over the subsequent 2 years following an acute coronary syndrome. It is a "positive" result - in favor of the concept of intensive statin therapy, but it was based on very strange study design.

2. the A to Z trial compared early intensive (40 mg/day of simvastatin for 1 month followed by 80 mg/day thereafter, n = 2,265) versus a delayed conservative strategy (receiving placebo for 4 months followed by 20 mg/day of simvastatin, n = 2,232) in patients with acute coronary syndromes. It was a "negative" study which did not achieve the prespecified end point.

3. The Treating to New Targets (TNT) trial: There were reductions in cardiovascular death, myocardial infarction (MI), need for revascularization, and stroke with use of high-dose vs. standard-dose atorvastatin. Although the trial results were consistent with the concept that for cholesterol, "the lower the better", concerns were raised regarding a nonsignificant difference in total and noncardiovascular death in favor of less intensive statins therapy. In other words, the most important issue for both patients and clinicians, a hard end-point -- total death -- moved in a wrong direction in this "positive" study.

4. The Incremental Decrease in End Points Through Aggressive Lipid Lowering (IDEAL) trial. In this study of patients with previous MI, intensive lowering of LDL-C (80 mg/day; n 4,439) did not result in a significant reduction in the primary outcome of major coronary events vs. usual-dose simvastatin (20 mg/day; n = 4,449). There were no differences in cardiovascular or all-cause mortality. Patients in the atorvastatin group had higher rates of drug discontinuation due to nonserious adverse events. "Negative" results.

So, out of the 5 trials which investigated intensive vs. standard statin regime, we have 2 "positive" with reservations (PROVE IT--TIMI 22 and TNT), 2 "negative" (A to Z and IDEAL) and 1 "missed" (SEARCH - suggestively negative). Alas, until now the bitterest comparator for the "intensive" statin monotherapy was 'usual-dose" statin monotherapy... then, in view of the lack of a proven effect of the current intensive "statin as monotherapy" strategy and "residual risk" concept, it is logical to ask whether other strategies could be more beneficial and safer [10].

### Fibrates: Evidences before the ACCORD Lipid study

Fibrates have been used in clinical practice for more than four decades due to their ability to substantially decrease triglyceride levels, increase HDL-C levels and in addition moderately but significantly reduce LDL-C [10]. Due to their beneficial effects on glucose and lipid metabolism, peroxisome proliferators-activated receptors (PPARs)-alpha agonists (fibrates) are good potential candidates for reducing the cardiovascular risk in subjects with atherogenic dyslipidemia typically present in the metabolic syndrome and type 2 diabetes [11-13].

Although less clinical intervention studies have been performed with fibrates than with statins, there were clear evidences that two of the fibric acid derivates -- gemfibrozil and bezafibrate -- reduce the risk of cardiovascular disease [14-20]. Interestingly, reduction of cardiovascular disease was more pronounced in patients displaying baseline characteristics very similar to metabolic syndrome definitions [14,15,21].

The primary-prevention trial Helsinki Heart Study (HHS) showed that treatment with gemfibrozil led to a significant reduction in major cardiovascular events [14]. In the Secondary prevention Veterans Affairs High-density lipoprotein cholesterol Intervention Trial (the VAHIT study) - which included 30% of diabetic patients -- gemfibrozil reduced the occurrence of major cardiovascular events by 22% [15]. Again, reduction of cardiovascular disease with gemfibrozil was more pronounced in patients displaying more than three of the features of metabolic syndrome [22,23]. The 18-year results from the Helsinki Heart Study shows that patients in the original gemfibrozil group had a 23% lower risk of CHD mortality compared with the original placebo group. Interestingly, those in the highest tertile of both body mass index and triglyceride level at baseline had the most dramatic risk reductions with gemfibrozil -- 71% (!) for coronary heart disease (CHD) mortality and 33% for all-cause mortality [24].

In the Bezafibrate Infarction Prevention (BIP) study an overall trend of a 9.4% reduction of the incidence of primary end point (fatal or nonfatal myocardial infarction or sudden death) was observed. The reduction in the primary end point in 459 patients with high baseline triglycerides (above 200 mg/dl) was significant [19]. Recent extension of the BIP trial demonstrated that patients with metabolic syndrome might be the ones to obtain the most marked benefit from therapy with fibrates [21,25-29]. Overall, bezafibrate treatment was associated with a reduced risk for fatal and nonfatal MI with hazard ratio (HR) and confidence interval (CI) of 0.71 (0.54-0.95) and 0.67 (0.49-0.91), respectively. The cardiac mortality risk tended to be lower on bezafibrate (HR 0.74, CI 0.54-1.03). Similarly to gemfibrozil Helsinki Heart Study extension, in patients with augmented features of metabolic syndrome [21] (at least 4 risk factors for metabolic syndrome) a marked (56%!) reduction in cardiac mortality was observed (HR 0.44, CI 0.25-0.80). In contrast, in patients without atherogenic dyslipidemia this favorable effect was absent: there was no significant difference in the cardiovascular end points between bezafibrate and placebo groups (for example, cardiac death was 7.7% vs. 7.7%). Also for fenofibrate, a post hoc analysis of the FIELD study suggested a benefit for patients with both elevated triglyceride levels and low HDL cholesterol levels [30]. A recent large meta-analysis [31] regarding the effects of fibrates on cardiovascular outcomes noted greater effect sizes in trials that recorded a higher mean baseline triglyceride concentration (p = 0.030). As expected, in a so called "general population" -- reflecting a blend of effects in patients with and without atherogenic dyslipidemia - a "mean diluted" effect of fibrate therapy was reduced, but still produceing a significant 10% relative risk (RR) decrease in major cardiovascular events (p = 0.048) and a 13% RR reduction for coronary events (p < 0.0001).

Therefore, a clear benefit of fibrate therapy did emerge among patients with atherogenic dyslipidemia (particularly high triglycerides and low HDL-C). This is the subgroup of patients for which fibrate treatment is indicated under current guidelines (exepting bezafibrate, which has been also used to treat biliary damage), this is the subgroup of patients in whom fibrates are typically prescribed in clinical practice and this is the subgroup of patients which should be investigated in the large clinical trial. So, it remains embarrassing that after all the achievements, mistakes and lessons of the previous fibrate's studies, an appropriate trial design has still not been utilized for fenofibrate.

### The ACCORD Lipid study: fibrate/statin combination in patients with atherogenic dyslipidemia

The researchers who planed the ACCORD Lipid study faced a conundrum when starting the trial [32], questioning whether it would be best to test the addition of fenofibrate to statin therapy in patients with dyslipidemia, in those with low HDL- C and high triglyceride levels (an appropriate population), or in a broader spectrum of patients (an inappropriate but larger population). Choosing the latter, they at least prespecified a number of subgroups, including patients with dyslipidemia. Unfortunately, patients with atherogenic dyslipidemia for whom fibrate is indicated represented less than 17 percent of the ACCORD Lipid population (941 out of 5518 patients), whereas in routine clinical practice the size of the problem is significantly greater. Anyway, in this subgroup analysis, the patients with higher baseline triglycerides and lower HDL-C levels benefited from fenofibrate therapy in addition to simvastatin, similarly to the previous fibrate's as monotherapy trials: the primary outcome rate was 12.4% in the fenofibrate group, versus 17.3% in the placebo group (28% crude HR reduction, CI less than 1, e.g. statistically significant findings). Among all other 4548 patients included in this analysis (patients without atherogenic dyslipidemia) such rates were 10.1% in both fenofibrate and placebo study groups.

Obviously, the epidemiological characteristics of ACCORD Lipid study depart from the real clinical practice: among people with type 2 diabetes, there is a high prevalence of atherogenic dyslipidemia and metabolic syndrome [33,34], which further accentuates cardiovascular risk. For example, an analysis of NHANES III data in adults aged ≥50 years showed that approximately 86% of patients with diabetes had also metabolic syndrome [33]. In this report, the prevalence of CHD was higher in those people with both diabetes and metabolic syndrome compared with those with diabetes alone (19.2% vs 7.5%). It was probably hard, but the researchers who planned ACCORD Lipid study succeeded to recruit a vast majority of patients with lipid profile uncommon in type 2 diabetes. Anyway, pooled together, evidence consistently demonstrated that fibrates offer optimum cardiovascular benefit in patients with atherogenic dyslipidemia. This dyslipidemia is typical for the metabolic syndrome and for most (but not all, as was confirmed by the ACCORD Lipid study researchers) patients with type 2 diabetes. In our point of view, the main finding of ACCORD Lipid study was the observation that fibrates may lead to cardiovascular risk reduction in patients with atherogenic dyslipidemia not only as monotherapy but in combination with statins as well.

## Conclusions

In patients with atherogenic dyslipidemia (high triglycerides and low HDL-cholesterol) fibrates both as monotherapy and as combination with statins were associated with reduced risk of cardiovascular events. In patients without dyslipidemia this favorable effect was absent: don't prescribe fibrates for these patients, please.

## List of Abbreviations

BIP: Bezafibrate Infarction Prevention study; CHD: coronary heart disease; HDL-C: high density lipoprotein cholesterol; LDL-C: Low density lipoprotein cholesterol; MI: myocardial infarction; PPARs: peroxisome proliferators-activated receptors.

## Competing interests

The authors declare that they have no competing interests.

## Author's contributions

Both authors have equally contributed in the conception and drafting of the manuscript.
